# Communication With American Indians and Alaska Natives About Cardiovascular Disease

**DOI:** 10.5888/pcd17.200189

**Published:** 2020-12-17

**Authors:** Amanda D. Boyd, Amber L. Fyfe-Johnson, Carolyn Noonan, Clemma Muller, Dedra Buchwald

**Affiliations:** 1 The Edward R. Murrow College of Communication, Washington State University, Pullman, Washington; 2 Elson S. Floyd College of Medicine, Washington State University, Seattle, Washington; 3 Partnerships for Native Health, Washington State University, Seattle, Washington

## Abstract

**Introduction:**

Cardiovascular disease (CVD) is the leading cause of death among American Indians and Alaska Natives. Reducing CVD risk requires effective communication about risk factors and preventive behaviors. Messages should be designed with an understanding of where people seek health information, their perceptions of a hazard, and their perception of information sufficiency. We examined these components of message design to inform strategies to effectively communicate information about CVD to American Indians and Alaska Natives.

**Methods:**

We surveyed 220 adults who self-identified as American Indians or Alaska Natives at 2 Native-focused events in urban areas. Our survey included items on demographic characteristics, place of residence, sources of information used to learn about CVD, perceived information sufficiency, and perceptions about the importance of CVD as a health problem.

**Results:**

Respondents used the internet (67%), their doctors (66%), friends and relatives (63%), brochures (62%), and television (61%) to learn about CVD. Participants aged 60 or older and those living on a reservation were more likely to use their doctor to learn about CVD than their younger (≤30 y) or urban peers. CVD was viewed as a major problem for American Indians and Alaska Natives (84%) and for Americans in general (86%). Most respondents felt moderately (54%) or well informed (37%) about CVD.

**Conclusion:**

Various information sources should be used to increase awareness about CVD. Special attention may be needed to optimize communication to American Indians and Alaska Natives aged 60 or older and people living on reservations. Further study is needed to determine how our findings can best inform effective interventions to reduce CVD morbidity and mortality among these populations.

**Summary**

**What is already known on this topic?**

Cardiovascular disease is the leading cause of death among American Indians and Alaska Natives. Reducing cardiovascular disease morbidity and mortality requires effective communication about cardiovascular health and preventive behaviors.

**What is added by this report?**

We surveyed participants during 2 Native-focused events to examine where people seek information about cardiovascular disease, their perceptions of the disease, and how informed they perceive themselves to be about cardiovascular disease.

**What are the implications for public health practice?**

By understanding factors that influence effective message design, culturally grounded communication strategies can be improved. Additional work is needed to determined how our findings can best inform effective interventions to reduce cardiovascular disease.

## Introduction

Cardiovascular disease (CVD) is the leading cause of death among American Indian and Alaska Native populations (1). The American Heart Association recently reported that these populations have a higher prevalence (12%) of CVD than people of Hispanic, non-Hispanic White, Black, and Asian race/ethnicity (range, 1%–8%) (2). Furthermore, American Indians have a disproportionate prevalence of CVD risk factors, including type 2 diabetes, hypertension, obesity, smoking, and hyperlipidemia than people of other races/ethnicities (3).

Health communication can raise awareness about health disorders, decrease misperceptions about risks, and increase behaviors that reduce risk for disorders or prevent them (4). Effective health communication with American Indians and Alaska Natives should consider cultural factors in designing messages and disseminating information (5) because of the distinctive values, traditions, knowledge, and languages of these groups (6). Health communication that does not account for cultural factors could contribute to health disparities (7).

Reducing CVD morbidity and mortality requires effective communication about cardiovascular health, modifiable CVD risk factors, and preventive behaviors (8). Effective message design requires an understanding of where people seek information, their perceptions of a health risk, and how informed they perceive themselves to be about a risk. The latter is also referred to as “perceived information sufficiency” (9). Understanding perceived information sufficiency can provide insight into message reception. For example, people who believe they are sufficiently informed about a risk may avoid additional communication about that risk (10). Despite the need for effective communication about CVD, virtually no research has examined where American Indians and Alaska Natives seek health information, their perceived information sufficiency about CVD, or their perceptions of the relative importance of CVD as a health problem. In the our study, we surveyed American Indian and Alaska Native adults at Native-focused events in 2 Washington State cities to learn where they obtained information about CVD, their perceptions about the relative importance of CVD as a health problem, and their perceived information sufficiency about CVD.

## Methods

### Data collection

We administered surveys in 2017 to 156 American Indians and Alaska Natives at the University of Washington Spring Powwow and to 64 American Indians and Alaska Natives at the National Tribal Health Conference. The University of Washington Spring Powwow, which in 2017 was held in Seattle, Washington, is an annual 2-day event that attracts more than 5,000 people. Attendees from numerous northwestern tribes (eg, Spokane, Tulalip, Yakima, Quinault, Skokomish) were represented at the powwows (11). Although the events are held in an urban environment, powwows draw American Indians and Alaska Natives from both urban and rural areas. The National Tribal Health Conference, hosted by the National Indian Health Board, was held in Bellevue, Washington, and comprised workshops, exhibitions, and more than 130 presentations from health service providers, community members, researchers, and government officials (12). Attendees at these 2 events are a rich cross section of people, ranging from tribal leaders and health officials to the lay public and students.

Partnerships for Native Health, a large research and education unit at Washington State University, hosted a booth at both events to administer a survey to identify health needs and to disseminate information on various health conditions common among American Indians and Alaska Natives. Attendees could approach the staff at the booth and learn about the survey. If interested, they were asked 2 screening questions to determine eligibility: 1) “Do you identify as American Indian or Alaska Native?” and 2) “Are you at least 18 years or older?” Respondents who completed the questionnaire were offered a $5 gift card in appreciation of their time and effort. All data were collected anonymously, and no identifying information was associated with the questionnaires. The study was determined to be exempt from review by the Washington State University institutional review board.

### Questions and measures

The questionnaire consisted of 10 questions on demographic characteristics, information seeking, perceptions of CVD risk, and perceived information sufficiency. Respondents were asked to indicate whether they had received (seen, heard, or read) information about CVD from any of the following sources: television, newspaper, radio, magazines, brochures, health professionals (including their doctor or other health professionals), friends and relatives, social media, or other internet sources. To examine perceptions about the relative importance of CVD as a health problem, respondents rated their level of agreement with 2 statements: “Heart disease is a large health problem for American Indians and Alaska Natives,” and “Heart disease is a large health problem for Americans in general.” Responses were on a 4-point scale from strongly disagree to strongly agree. We computed a measure to compare respondents’ perceptions about CVD among American Indians and Alaska Natives compared with their perceptions about CVD among Americans in general by calculating the difference in agreement between the 2 statements. Differences of 0 indicated a respondent had the same level of agreement on CVD as a health problem for both groups. Differences of −3 to −1 indicated a respondent showed more agreement that CVD was a health problem for American Indians and Alaska Natives. Differences of 1 to 3 indicated a respondent showed more agreement that CVD was a health problem for Americans in general. To assess perceived information sufficiency, respondents were asked “How informed are you about heart disease?” Responses were measured on a 4-point scale from very well informed to not at all informed. This question was derived from a study that examined health perceptions and CVD, part of the Dallas Heart Study, which was a longitudinal study of cardiovascular health among Dallas County adults (13). The demographic characteristics we collected were age in years, sex, and education. Age was collected as a continuous variable but categorized as 18 to 29, 30 to 39, 40 to 49, 50 to 59, and 60 to 77 years. Response options to indicate participant sex included female, male, and other. Education was categorized as less than high school, high school graduate/general equivalency diploma/vocational school diploma, some college, or college graduate. Location of residence was determined by asking “Where do you live most of the year?” Response options were a reservation, a rural town or area but not on a reservation, or a large metropolitan area.

### Data analysis

We calculated descriptive statistics as mean and range for continuous variables and number and percentage for categorical variables. We used Poisson and multiple logistic regression models to examine differences in information sources used to learn about CVD, perceptions of the relative importance of CVD as a health problem, and perceived information sufficiency. All models included independent variables for age, sex, education, and residence. Tests for differences according to age and education were assessed by using a test for trend; we used an omnibus Wald test to assess differences according to sex and residence. Regression results are presented as prevalence ratios and 95% confidence intervals or as average adjusted predictions and 95% confidence intervals calculated from the models by using Stata’s margins command (14). All analyses were conducted using Stata 15.1 (StataCorp). Significance was established at the α = .05 level, although we also interpreted 95% CIs as representing a wider range of plausible true parameters.

## Results

The mean age of the 220 respondents was 44 (range, 18–77); almost three-quarters identified as female (72%), and most had completed at least some college or were college graduates (74%) (Table 1). Nearly half of respondents lived in a large metropolitan area (45%).

**Table 1:** Demographic Characteristics of American Indian and Alaska Native Adults (N = 220) Who Attended an Event in 2017^a^

Characteristic	University of Washington Powwow, n (%)	National Tribal Health Conference, n (%)	Total, N (%)
**Age, y**			
18–29	33 (22)	2 (3)	35 (16)
30–39	36 (24)	15 (23)	51 (24)
40–49	28 (18)	17 (27)	45 (21)
50–59	33 (22)	16 (25)	49 (23)
60–77	23 (15)	14 (22)	37 (17)
**Sex**			
Female	108 (69)	51 (80)	159 (72)
Male	48 (31)	13 (20)	61 (28)
**Education**			
Less than high school	10 (6)	0 (0)	10 (5)
High school/GED/vocational school	39 (25)	9 (15)	48 (22)
Some college	58 (38)	23 (37)	81 (38)
College graduate	47 (31)	30 (48)	77 (36)
**Residence**			
Reservation	30 (20)	38 (59)	68 (31)
Rural area or town	37 (24)	15 (23)	52 (24)
Metropolitan area	86 (56)	11 (17)	97 (45)

Abbreviation: GED, general equivalency diploma.

a Survey was administered to 156 American Indians and Alaska Natives at the University of Washington Spring Powwow and to 64 at the National Tribal Health Conference. Total sample sizes may not equal 220 because of missing values. Percentages may not total 100 because of rounding.

**Figure 1:**
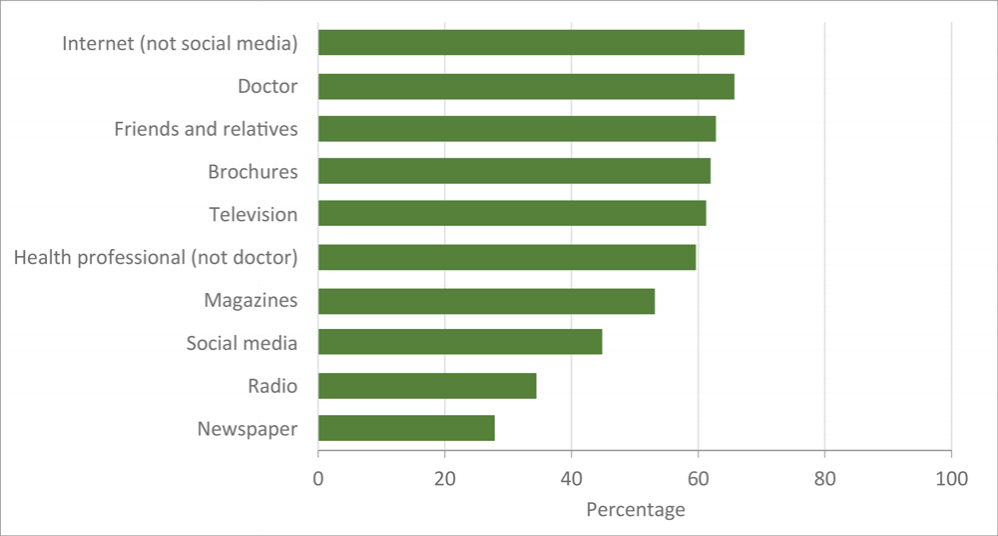
Information sources used by American Indians and Alaska Natives (N = 220) to learn about cardiovascular disease.

More than half of respondents indicated using the internet (67%), their doctor (66%), friends and relatives (63%), brochures (62%), and television (61%) to learn about CVD (Figure 1), with no differences according to sex (Table 2). Participants with more education were more likely to use the internet (prevalence ratio [PR] = 1.3; 95% CI, 0.9–1.7 for college graduates vs high school graduates). Older participants (PR = 1.5; 95% CI, 1.0–2.3 for age ≥60 vs 18–29), and those living on a reservation (PR = 1.4; 95% CI, 1.1–1.8 for reservation vs metropolitan area) were more likely to use their doctor to learn about CVD.

**Table:** 

Information source	%
Internet (not social media)	67
Doctor	66
Friends and relatives	63
Brochures	62
Television	61
Health professional (not doctor)	60
Magazines	53
Social media	45
Radio	34
Newspaper	28

**Table 2:** Sources of Information About Cardiovascular Disease Among Survey Respondents, By Demographic Characteristics^a^

Characteristic	Information Source				
	Internet (n = 196)	Doctor (n = 201)	Friends/Relatives (n = 198)	Brochures (n = 196)	Television (n = 200)

	% (95% CI)	% (95% CI)	% (95% CI)	% (95% CI)	% (95% CI)
**Overall**	67 (61–74)	66 (59–72)	62 (56–69)	62 (55–69)	62 (54–67)
**Age, y**					
18–29	76 (61–91)	50 (31–69)^b^	64 (48–80)	34 (17–50)	79 (63–94)
30–39	58 (44–71)	58 (44–71)	65 (52–79)	67 (57–82)	58 (44–72)
40–49	66 (52–80)	69 (55–82)	53 (38–68)	70 (56–85)	59 (44–74)
50–59	77 (65–89)	74 (60–87)	67 (53–82)	68 (55–82)	58 (43–72)
60–77	56 (38–75)	77 (64–90)	58 (40–75)	59 (41–76)	57 (40–74)
**Sex**					
Female	66 (59–74)	64 (57–72)	62 (54–70)	64 (56–71)	63 (55–71)
Male	68 (57–80)	70 (56–83)	61 (48–74)	56 (43–69)	58 (45–71)
**Education**					
Less than high school graduate	36 (2–70)	76 (46–100)	60 (25–95)	61 (31–91)	75 (47–100)
High school graduate/GED/vocational school diploma	56 (42–71)	63 (50–77)	60 (45–75)	58 (43–73)	65 (51–78)
Some college	71 (61–81)	69 (58–79)	66 (56–77)	59 (48–69)	64 (53–75)
College graduate	72 (61–83)	63 (52–74)	58 (46–69)	67 (56–78)	55 (43–68)
**Residence**					
Reservation	63 (51–75)	78 (68–89)^c^	58 (46 −71)	65 (54–75)	64 (51–77)
Rural area or town	61 (47–75)	67 (53–80)	72 (59–85)	65 (51–78)	69 (57–82)
Metropolitan area	72 (63–81)	56 (46–66)	58 (48–68)	58 (48–68)	56 (46–66)

a Estimates and tests adjusted for age, sex, education, and residence (except overall).

b *P* value = .004, test for trend with age from Poisson regression model including age, sex, education, and residence as independent variables.

c *P* value = 0.02, omnibus Wald test for residence from Poisson regression models including age, sex, education, and residence as independent variables.

**Figure 2:**
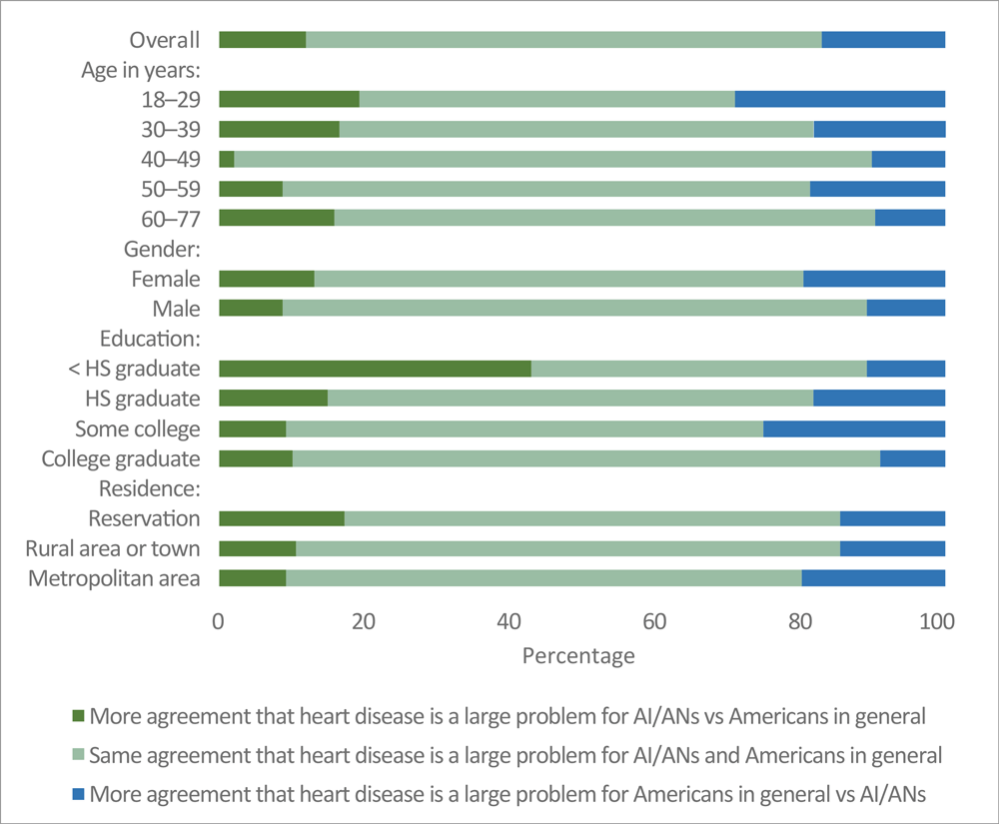
Comparison of agreement among American Indians and Alaska Natives that cardiovascular disease is a larger problem for them than for Americans in general, by respondent characteristics. Abbreviations: AI/ANs, American Indians/Alaska Natives; HS, high school.

Most participants agreed that CVD was a large problem for both American Indians and Alaska Natives (84%) and for Americans in general (86%) (Table 3). Perceptions of CVD as a health problem did not differ by age, sex, education, or residence. Most participants had the same level of agreement on CVD as a health problem for both groups (71%; 95% CI, 65%–77%, Figure 2). However, 12% (95% CI, 8%–16%) of participants showed more agreement that CVD is a larger problem for American Indians and Alaska Natives than for Americans in general, and 17% (95% CI, 12%–22%) showed more agreement that CVD is a larger problem for Americans in general than for American Indians and Alaska Natives. The percentage of participants who perceived the same CVD risk for both groups was higher among men than women (PR = 1.2; 95% CI, 1.0–1.4), among respondents aged ≥60 than among respondents aged <30 (PR = 1.4; 95% CI, 1.0–2.0 ), and among college graduates than among high school graduates (PR = 1.5; 95% CI, 0.9–2.7).

**Table 3:** Perception of Cardiovascular Disease Risk and Perceived Information Sufficiency Among Survey Respondents, By Demographic Characteristics^a^

Characteristic	Cardiovascular Disease Risk Perception		Perceived Information Sufficiency
	Large Health Problem for AI/ANs^b ^(n = 207)	Large Health Problem for Americans^b ^(n = 207)	Very Well or Well Informed (n = 202)
**Overall**	84 (78–89)	86 (81–90)	37 (30–44)
**Age, y**			
18–29	85 (72–98)	90 (80–100)	32 (18–46)
30–39	79 (67–90)	78 (66–90)	33 (22–44)
40–49	95 (89–100)	94 (88–100)	45 (29–61)
50–59	78 (66–90)	82 (71–92)	38 (25–52)
60–77	84 (72–96)	89 (79–100)	39 (21–56)
**Sex**			
Female	83 (77–89)	86 (80–91)	37 (30–44)
Male	86 (76–95)	87 (79–96)	37 (25–50)
**Education**			
Less than high school	89 (67–100)	97 (91–100)	11 (0–31)^c^
High school/GED/ vocational school	78 (66–89)	75 (62–87)	30 (16–44)
Some college	86 (79–94)	90 (84–97)	26 (17–36)
College graduate	85 (77–93)	87 (80–95)	57 (45–68)
**Residence**			
Reservation	88 (80–96)	82 (73–91)	25 (15–34)^d^
Rural area or town	85 (74–95)	91 (82–99)	38 (24–52)
Metropolitan area	81 (73–89)	86 (79–93)	46 (36–57)

Abbreviation: AI/AN, American Indian and Alaska Native; GED, general equivalency diploma.

a Estimates and tests adjusted for age, sex, education, and residence (except overall). Values are percentage (95% CI).

b Strongly agree or agree.

c *P* = .001, test for trend with education from Poisson regression models including age, sex, education, and residence as independent variables.

d *P* = .03, omnibus Wald Test for residence from Poisson regression models including age, sex, education, and residence as independent variables.

**Table:** 

Characteristic	More Agreement That Heart Disease Is a Large Problem for AI/ANs than Americans in General	Same Agreement That Heart Disease Is a Large Problem for Both AI/ANs and Americans in General	More Agreement That Heart Disease Is a Larger Problem for Americans in General than AI/ANs
**Overall**	12	71	17
**Age, y**			
18–29	19	52	29
30–39	17	65	18
40–49	2	88	10
50–59	9	72	19
60–77	16	74	10
**Sex**			
Female	13	67	20
Male	9	80	11
**Education**			
<High school graduate	43	46	11
High school graduate	15	67	18
Some college	9	66	25
College graduate	10	81	9
**Residence**			
Reservation	17	68	15
Rural area or town	11	75	15
Metropolitan area	9	71	20

More than one-third of respondents (37%) reported they were well or very well informed about CVD (Table 3), 54% said they were moderately well informed, and 9% said they were not at all informed. Perceived information sufficiency was similar according to age and sex, but higher percentages of participants who were college graduates perceived themselves as well informed than those who had completed less education. Similarly, participants living in large metropolitan areas were more likely to perceive themselves as well informed about CVD than their counterparts living on a reservation.

## Discussion

Use of effective communication channels is key to educating a population about health risks and can improve peoples’ health and well-being (15). To understand what communication channels should be used to disseminate health information requires knowledge of where people seek information (9). We found that American Indians and Alaska Natives used numerous communication channels, including the internet, doctors, friends and relatives, brochures, and television, to find information about CVD. These results are consistent with other studies that examined health information–seeking behaviors among minority populations (16,17). In addition, preferred information sources varied by demographic characteristics. Respondents aged 18 to 30 commonly used the internet, and those aged 60 or older or living on reservations used interpersonal sources, such as doctors. Such variations are consistent with research in other populations (4) and underscore the need for targeted and effective communication strategies for population subgroups (18). Providing information to elders during doctor visits but using internet messaging with younger people could optimize effective communication and health messaging among American Indian and Alaska Native populations.

We also examined perceptions of CVD as a problem for Americans in general and for American Indian and Alaska Native populations specifically. Knowledge of a population’s perceptions of a hazard can guide public health programs and communication strategies (19). If people are not concerned about a risk, they are unlikely to take steps to prevent it (20). Although accurate perceptions about health issues do not always translate to behavior change, having accurate perceptions of a risk is postulated to be a prerequisite for positive health behaviors (21). Thus, if CVD is not perceived as a problem, American Indians and Alaska Natives may be unlikely to engage in regular exercise, control their blood pressure, or make other lifestyle changes to reduce their CVD risk. In our study, most respondents accurately identified CVD as a major health problem for both the general US population (86%) and for American Indians and Alaska Natives specifically (84%).

Our findings contribute to the scant existing literature on perceived CVD risk and perceived information sufficiency among American Indians and Alaska Natives. In a 2003 study, 516 American Indians in Montana were asked about their history of CVD, risk factors, and their perceived risk of CVD (22). Participants reported a high prevalence of CVD risk factors, and most recognized the risks associated with modifiable factors. Although our study showed a higher percentage of respondents identifying CVD as an important problem than that study, we did not ask respondents to self-report CVD conditions and could not evaluate potential differences in perception on the basis of health status. Another study examined CVD knowledge and perceptions of risk among 22 American Indian women with gestational diabetes (23). Most of the women had high levels of knowledge about CVD and believed their risk was high, yet they reported low self-efficacy related to preventing CVD. Collectively, these studies suggest that although education is important, health communication campaigns must be integrated into more comprehensive interventions to reduce CVD risk among American Indian and Alaska Native populations. We were unable to locate other published studies attempting to characterize perceived CVD risk or perceived information sufficiency among these heterogeneous populations, underscoring the need for more research.

In the few empiric studies that we found of knowledge and awareness of CVD among American Indian and Alaska Native populations, knowledge was relatively high (24). In a survey of 298 urban American Indians and Alaska Natives, most respondents were aware of the risks associated with a high-salt diet, saturated fats, overweight and obesity, and secondhand smoke and of the benefits of physical activity (25). However, many respondents did not know whether a specific blood pressure value was considered high. Additional studies identified differences in CVD knowledge among subgroups of these populations. For example, in a study in which American Indian participants in the Strong Heart Study were asked whether 9 known risk factors affected a person’s chances of having CVD, the 2 most consistent variables associated with greater heart disease knowledge were female sex and higher education (26). In another study examining CVD knowledge among American Indian, Hispanic, and African American populations, the 21 American Indian women in the study had the lowest baseline knowledge of CVD (27).

In combination with knowledge of CVD, exploring perceived information sufficiency is essential for effective health communication and message design and dissemination (9). Perceived information sufficiency can provide an indication of how people will process and attend to the information. People with low perceptions of information sufficiency may be more likely to seek risk-related information and more likely to process it systematically (eg, consider diverse views and how they may apply to their own lives) than those with high perceptions of information sufficiency (10). Most respondents in our study believed they were moderately informed about CVD, and only 8% felt very well informed. These results are consistent with other studies examining perceived information sufficiency among other populations. For example, in a study of 1,000 American women (66% White, 13% African American, 13% Hispanic), less than 20% in each group responded that they were well-informed about CVD (18). Our results, and those of others (18), suggest that people may be open to receiving new information about CVD. It also highlights the need for public health officials to effectively communicate about CVD to ensure greater perceived information sufficiency.

We also found that perceived information sufficiency varied by demographic factors, such as age and location of residence. Our findings are consistent with other research showing that people who are highly educated are more likely to feel knowledgeable or have adequate information about a health topic (10,28). Likewise, we noted that participants living in large metropolitan areas were more likely to perceive themselves as well informed about CVD than those living on a reservation. Special attention may be needed to optimize communication to American Indians and Alaska Natives living on reservations or in other rural locations. In this regard, studies have highlighted the benefits of interventions and curricula aimed at increasing knowledge of CVD among these groups. In a small randomized trial evaluating a program to deliver education about CVD and diabetes, American Indian and Alaska Native participants who received the Honoring the Gift of Heart Health curriculum improved their knowledge about heart attacks and marginally improved their general knowledge about CVD (24). Another study examining outcomes of a clinic-based educational intervention for CVD prevention among minority populations demonstrated improved knowledge of CVD among American Indians (27). Taken together, these studies indicate that education can improve knowledge of CVD, but further study is needed to determine whether the results lead to preventive behaviors that reduce CVD morbidity or mortality.

Our study had limitations. First, we surveyed a convenience sample that is unlikely to be representative of the larger American Indian/Alaska Native population in our region. Nearly three-quarters of our sample were women, and most had completed at least some college or were college graduates. However, population-based samples are difficult to access, especially in urban settings. American Indians and Alaska Natives do not live in defined neighborhoods or areas and cannot be identified by surnames. Tribal rolls, which enumerate all tribe members, are not usually available to researchers. Nevertheless, our study sample was recruited from 2 large, very different cultural events that drew American Indians and Alaska Natives from across the Pacific Northwest and beyond; therefore, our findings may pertain to a larger population than a study that is limited to a single reservation community. These hypothesis-generating findings could stimulate research in larger, more population-representative samples. Second, more than one person in the same family or household may have completed our survey, but that information was not collected and could not be accounted for in our analyses. Because of potential correlations between family member or household responses, CIs may be too narrow. Third, we examined perceptions about the relative importance of CVD as a health problem for American Indians and Alaska Natives specifically and for Americans in general. Examining general perceptions provides insight into the subjective judgements that people make about a risk and helps determine what people care about, which in turn informs risk communication strategies (9). In contrast, examining general risk perceptions does not provide insight into individual risk perceptions or understanding of whether participants feel that they themselves are at risk of CVD. Similarly, we did not collect data on previously diagnosed CVD, and we could not evaluate whether perceptions differed among our respondents with and without prevalent disease. Questions related to risk perceptions and information sufficiency have not been previously validated among American Indian and Alaska Native populations. However, our study begins to provide insight into risk perceptions among these populations and begins to illuminates avenues for effective, culturally appropriate communication about CVD.

Our study illustrates the importance of conducting population-based research to more conclusively understand where and how American Indians and Alaska Natives seek information about cardiovascular health, their perceptions of the importance of CVD as a health problem, and their perceived and actual CVD information sufficiency. Monitoring trends in risk perceptions and information requirements can help determine whether communication strategies should change to meet the needs of the target audience (28). Research can elucidate whether messages delivered through communication channels that have been proven effective and that are tailored to the needs of different demographic urban and rural subgroups can effectively inform American Indians and Alaska Natives about cardiovascular health and, ultimately, help reduce their CVD morbidity and mortality.
